# Concurrent Methane Production and Oxidation in Surface Sediment from Aarhus Bay, Denmark

**DOI:** 10.3389/fmicb.2017.01198

**Published:** 2017-06-30

**Authors:** Ke-Qing Xiao, Felix Beulig, Kasper U. Kjeldsen, Bo B. Jørgensen, Nils Risgaard-Petersen

**Affiliations:** Center for Geomicrobiology, Department of Bioscience, Aarhus UniversityAarhus, Denmark

**Keywords:** methanogenesis, methane oxidation, surface sediment, methanogenic archaea, isotope dilution

## Abstract

Marine surface sediments, which are replete with sulfate, are typically considered to be devoid of endogenous methanogenesis. Yet, methanogenic archaea are present in those sediments, suggesting a potential for methanogenesis. We used an isotope dilution method based on sediment bag incubation and spiking with ^13^C-CH_4_ to quantify CH_4_ turnover rates in sediment from Aarhus Bay, Denmark. In two independent experiments, highest CH_4_ production and oxidation rates (>200 pmol cm^-3^ d^-1^) were found in the top 0–2 cm, below which rates dropped below 100 pmol cm^-3^ d^-1^ in all other segments down to 16 cm. This drop in overall methane turnover with depth was accompanied by decreasing rates of organic matter mineralization with depth. Molecular analyses based on quantitative PCR and MiSeq sequencing of archaeal 16S rRNA genes showed that the abundance of methanogenic archaea also peaked in the top 0–2 cm segment. Based on the community profiling, hydrogenotrophic and methylotrophic methanogens dominated among the methanogenic archaea in general, suggesting that methanogenesis in surface sediment could be driven by both CO_2_ reduction and fermentation of methylated compounds. Our results show the existence of elevated methanogenic activity and a dynamic recycling of CH_4_ at low concentration in sulfate-rich marine surface sediment. Considering the common environmental conditions found in other coastal systems, we speculate that such a cryptic methane cycling can be ubiquitous.

## Introduction

Methane (CH_4_) generation by methanogenic archaea is the main terminal step in organic matter mineralization in marine sediments depleted of electron acceptors for microbial respiration (Reeburgh, 2007). Three major methanogenic pathways are known based on the type of carbon sources catabolized, i.e., hydrogenotrophic methanogenesis using CO_2_ and H_2_, acetoclastic methanogenesis using acetate, and methylotrophic methanogenesis using methylated compounds, such as methanol, methylamines (MAs) or methyl sulfide [dimethyl sulfide (DMS), dimethylsulfoniopropionate (DMSP)] (Whitman et al., 2006). In the presence of sulfate, sulfate-reducing bacteria (SRB) usually outcompete methanogens for common substrates like H_2_ and acetate due to their higher substrate affinity and higher energy yield (Schönheit et al., 1982; Lovley and Klug, 1983). Microbial CH_4_ production is usually detected in the sub-surface part of marine sediments, where sulfate is depleted. However, as a range of methylated compounds can be used by methylotrophic methanogens but generally not by SRB, methanogenesis from these non-competitive substrates is still feasible in surface sediments with high sulfate concentration (Reeburgh, 2007). Such surface sediments receive an influx of detrital organic matter containing different types of molecules, such as lignin, pectin, choline, creatine, and betaine, which can provide a source of methylated substrates for methanogenesis (Oremland et al., 1982; King, 1984; Kiene et al., 1986). Thus, the distribution and activity of methanogens in marine sediments may be controlled not only by competition with SRB for H_2_ and acetate, but also by the availability of these methylated compounds. In addition to being a metabolic end product, CH_4_ is also an energy substrate to sulfate, nitrate, iron, and oxygen-respiring microorganisms in shallower sediment layers (Reeburgh, 2007; Knittel and Boetius, 2009; Ettwig et al., 2010, 2016). The upward diffusive flux of CH_4_ from the methanogenic zone is typically consumed in shallow layers, and CH_4_ rarely escapes the seafloor (Knittel and Boetius, 2009; Valentine, 2011). Although many studies have suggested that methanogenesis can occur in sulfate-rich environments (Oremland et al., 1982; Mitterer et al., 2001; Mitterer, 2010; Maltby et al., 2016; Zhuang et al., 2016), the magnitude, distribution and pathways of this production remain poorly constrained.

In this study, we quantified concurrent CH_4_ production and consumption rates using an isotope dilution method based on sediment bag incubation. A previous study showed a downcore distribution of trimethylamine (TMA), DMS, and DMSP concentrations in sediment from the same site in Aarhus Bay, which all peaked in the top few centimeters, and supported the potential for methane generation through the methylotrophic pathway (Zhuang, 2014). We therefore hypothesized that this should be reflected in the downcore distribution of CH_4_ production in surface sediment of Aarhus Bay, and that the methanogens involved were dominated by methylotrophic groups. As CH_4_ concentration profiles rarely indicate methanogenesis in surface sediments, we further hypothesize that a cryptic methane cycle exists here in which CH_4_ production and oxidation is tightly coupled. Such a new prospect of CH_4_ turnover may challenge current knowledge of carbon cycling in sulfate-rich sediments.

## Materials and Methods

### Location and Sampling

The study was performed in Aarhus Bay where organic-rich Holocene marine mud has accumulated up to >10 m thick layers on top of the glacial deposits over the past 8000 years (Flury et al., 2016). The sedimentation rate at station M5 (56°06.20°N and 10°27.47°E; water depth 28 m), based on ^14^C-dating of mollusk shells, has been stable for the last 4300 years (Langerhuus et al., 2012). The water column is generally oxic, oxygen penetration depth in the sediment is low (1–5 mm; Rasmussen and Jørgensen, 1992), and bioturbation is visible from sediment color and faunal tracks in the top 3 cm. Sediments for incubation were sampled in December 2015 and May 2016 with a Haps corer (Kanneworff and Nicolaisen, 1972), then transferred into a gravity core liner (diameter 11.8 cm) on deck, brought back to the laboratory, kept at *in situ* temperature, and processed within 48 h. The temperature of the bottom water, measured by thermometer, was around 10°C at both sampling times and the salinity was 30‰, determined by a handheld refractometer. Rumohr Lot cores (Meischner and Rumohr, 1974) were taken in parallel for geochemical profiles and molecular analysis of the methanogen community, and the sampling intervals were 2 cm in the top 20 cm. Sediment samples for molecular analysis were taken with sterile 5 ml cut-off plastic syringes and preserved at -80°C. Pore-water samples for sulfate and dissolved inorganic carbon (DIC) analysis were extracted from the intact sediment core via Rhizon suction samplers (0.1 μm porous polymer, Rhizosphere Research, Wageningen, Netherlands), pretreated as described previously (Glombitza et al., 2014). Pore-water samples for DIC were kept at 4°C before analysis. For sulfate, the extracted pore-water was flushed with CO_2_ to remove hydrogen sulfide, and frozen at -20°C until analysis. For methane sampling, 3 ml of water close to the sediment surface (< 1 cm) was taken with a 20 ml syringe mounted with a long PVC tube, and 2 ml of sediment was collected with cut-off plastic syringes. All samples were transferred immediately to 10 ml glass serum vials (preloaded with 4 ml distilled water and 1.5 g NaCl), which subsequently were closed with a butyl rubber septum and a crimp cap. The serum vials containing sediment were shaken, and preserved at -20°C before analysis.

### Diffusive Fluxes of CH_4_

Diffusive fluxes of CH_4_ were estimated from concentration gradients using Fick’s first law:

$$J=-\varphi /(1-In\left(\varphi^{2}\right))\times D\times \partial C/\partial z$$

where *J* is the diffusive flux of CH_4_, *C* is the concentration, *z* is depth (cm), and φ is porosity (Boudreau, 1997). *D* is the diffusion coefficient in free solution for a salinity of 30 and temperature of 10°C: D_CH4_ = 1.02 cm^2^ d^-1^ (Dale et al., 2008). The factor 1 - ln(φ^2^) corrects for the lower diffusion coefficient in sediment due to tortuosity (Boudreau, 1997).

### Bag Incubation Setup and Gas-Tightness Test

Plastic bags made of gas-tight multi-laminar plastic film (Supplementary Figures S1a, b) (Amcor, Denmark) were used for sediment incubation and ^13^CH_4_ spiking experiments. This bag incubation method was developed by Hansen et al. (2000), but some modifications were made: (1) the bags were sealed with a black slide binder (LEITZ, Germany) on one side, (2) the silicon gaskets were replaced by butyl rubber gaskets to improve gas tightness (Supplementary Figure S1c), (3) the plastic bags were mounted with a glass outlet custom made to fit the cut-off 2.5 ml syringes (Supplementary Figure S1d), (4) to physically protect the incubation bags and minimize oxygen penetration, they were placed in an outer gas-tight bag filled with N_2_. Prior to the isotope spiking experiments, gas-tightness tests were conducted by filling bags with artificial sea water brine of 300‰ salinity (for inhibiting microbial activities). CH_4_-saturated artificial sea water was added to two parallel bags, leaving no headspace, and incubated at 15°C. Triplicate samples were taken by syringes with a needle through the stopper and transferred into 3 ml glass vials. Methane in the water samples was measured by the headspace method using gas chromatography (SRI Instruments, United States). Gas-tightness tests were conducted by monitoring CH_4_ concentration in the bags over time. Within 140 days, methane concentrations in the bag were very stable with little fluctuations (ANOVA, *P* > 0.05; Supplementary Figure S2), suggesting that this type of bags can be used for incubation experiments over many days or even months. By this incubation method, sediment is kept in closed bags free of air and headspace and without dilution or stirring. This maintains an environment close to the *in situ* conditions (Hansen et al., 2000), which is critical for the study of natural process rates.

### Bag Incubation and Spiking with ^13^C-Labeled Methane

The following manipulations were all done under strict anoxic conditions in a glove box (Plas-Lab, Inc., United States), which was continuously flushed with nitrogen gas. Oxygen concentration in the glove box was <0.5 μmol l^-1^, as monitored with an oxygen sensor (Revsbech, 1989). Sediments from Haps cores were sliced into six 2 cm segments, five in the top 10 cm, and one around 15 cm. The sliced segments were passed through a 2 mm sieve to remove macrofauna and hard objects such as carbonate shells which could puncture the bag, and then loaded into the gas-tight bag, carefully avoiding headspace and removing gas bubbles. After 2–3 days of stabilization, ^13^C-labeled methane was introduced into each bag by injecting 20–100 μL of ^13^CH_4_-containing artificial sea water (excluding sodium sulfate), which resulted in a ^13^C isotope enrichment of ∼2% with minimal change (∼2%) of the total CH_4_ concentrations in pore-water. The sediment was homogenized by manual kneading of the bag for 20 min (Hansen et al., 2000). Six bags with sediment were incubated at 10°C in a thermostated incubator (MMM Incucell, Germany). At each sampling time, triplicate samples were taken by squeezing the bag to push the sediment through the glass outlet, where a PVC tube connected the outlet to a cut-off 2.5 ml syringe to ensure tightness (Supplementary Figure S1d). Sediment collected into the syringe was immediately transferred into 10 ml glass serum vials, which were closed with a butyl rubber septum and crimp cap, and preserved at -20°C, as described above.

Methane production and oxidation were calculated from the CH_4_ concentration and its isotope composition using an isotope dilution model, which was originally developed to determine rates of NH_4_^+^ turnover in anoxic marine sediments (Blackburn, 1979). The model here presumes that the concentration of CH_4_ changes during the experiment through time due to constant methane consumption (i.e., oxidation) (r) and production (p), thus:

$$Ct=C_{0}+(p-r)t$$

The model further presumes that the mole fraction of ^13^CH_4_ [R_t_ = ^13^CH_4_/(^13^CH_4_ + ^12^CH_4_)] changes trough time as a result of production of CH_4_ from indigenous sources and consumption of CH_4_ due to anaerobic oxidation in the sediment. In case the rate of methane production, p, is different from rate of methane consumption, r, the relationship between R_t_ and time can be described with the following expression:

$$In(R_{t}-R_{b})=In(R_{0}-R_{b})-\left(p/\left(p-r\right)\right)In(C_{t}/C_{0})$$

where R_b_ is the mole fraction of ^13^CH_4_ in CH_4_ being produced from indigenous sources in the sediment and R_0_ is the mole fraction of ^13^CH_4_ at the start of the experiment (see SI for the deduction of Eq. 2, and the equation when p = r). Equation 1 shows that the quantity “p - r” equals the slope of a regression line obtained from plotting C_t_ against, while Eq. 2 shows that the quantity “-p/(p - r)” equals the slope of a regression line obtained from plotting ln(R_t_-R_b_) against ln(C_t_/C_0_). Therefore p and r can each be calculated from the combination of these slopes.

### Mineralization of Organic Matters

The mineralization rates were measured by two methods, (1) during both cruises, sulfate reduction rates (SRRs) were determined the using the whole-core injection method (Jørgensen, 1978). Two microliters of carrier-free ^35^S-sulfate solution containing approximately 100 kBq (ca. 2.5 μCi) of radioactivity, were injected in 2 cm intervals at multiple depths in 26-mm-i.d. core. The formation of radiolabeled products was analyzed after incubation for 12–19 h at the *in situ* temperature. Incubations were terminated by sectioning the core into 2 cm segments that were fixed in weighed vials containing 10 ml of cold 5% Zn-acetate. The sediment was immediately mixed and stored at -20°C up to 4 weeks until further analysis. After thawing, the total reduced inorganic sulfur (TRIS = H_2_S, S_0_, FeS, and FeS_2_) was separated from the ^35^SO_4_^2-^ by a single step cold chromium distillation (Kallmeyer et al., 2004). The radioactivity of ^35^SO_4_^2-^ and ^35^S-TRIS was counted in 15 ml scintillation cocktail (Gold Star, Meridian Biotechnologies, United Kingdom) on a TriCarb 2900TR liquid scintillation analyzer (Packard Instrument Company, Germany). The *ex situ* SRRs were then calculated accordingly with a correction factor of 1.06 (Jørgensen, 1978; Flury et al., 2016); (2) In May 2016, accumulation of ammonium (NH_4_^+^) and DIC were also monitored in the bags to estimate mineralization rates. Sediment subsamples of 2.5 ml were taken from each bag and centrifuged, NH_4_^+^, DIC, and calcium were analyzed in the supernatant. The Ca^2+^ concentrations in sediment during the incubation were quite stable with little change over time (ANOVA, *P* > 0.05). The production rates of NH_4_^+^ and DIC were calculated by plotting concentrations against time and using linear fitting, and production rates of NH_4_^+^ were multiplied by a factor of 1.3 to correct for adsorption, according to Canfield et al. (1993a). The rates of organic carbon mineralization rates from SRR were calculated with a 1:2 stoichiometry between SRR and organic C oxidation (Flury et al., 2016).

### Chemical Analysis

Methane was measured using a Gas Chromatograph (GC) (310C, SRI Instruments, United States) with a flame ionization detector. The carbon isotope composition of methane was determined using a coupled pre-concentration GC/Isotope Ratio Mass Spectrometer system (GC/IRMS) (Thermo Fisher Scientific, Germany) (Rice et al., 2001). The δ^13^C values were reported vs. the Vienna Pee Dee Belemnite standard (VPDB). DIC was measured on the same GC/IRMS system without pre-concentration (Torres et al., 2005). Sulfate was measured by an IC-2500 ion chromatography system (Dionex Corporation, United States), NH_4_^+^ was determined by a FLUOstar Omega spectrophotometer (BMG LABTECH, Germany) based on the salicylate-hypochlorite method (Bower and Holm-Hansen, 1980), and calcium was measured by inductively coupled plasma optical emission spectrometry (ICP-OES) (Perkin Elmer Optima 2000DV, United States).

### DNA Extraction, Amplification, and Sequencing

Aliquots of frozen sediment samples (0.2 g) from the parallel Rumohr Lot core were used for total DNA extraction. Sediment samples were thawed at room temperature in a lysis buffer mixture containing 0.65 ml sodium phosphate buffer solution (112.9 mM Na_2_HPO_4_, 7.1 mM NaH_2_PO_4_) and 0.2 ml SDS solution (500 mM Tris–HCl, 100 mM NaCl, 10 w.-% sodium dodecyl sulfate, pH 8.0), as well as 0.25 ml zirconia beads (0.1 mm diameter, BioSpec, United States). The thawed mixture was subjected to bead beating at 50 oscillations s^-1^ for 1 min using a TissueLyser LT 2500 (Qiagen, Germany), followed by incubation in a thermomixer with 600 rpm at 50°C. After lysis, the mixture was centrifuged for 10 min at 19,000 × *g* at 4°C. The supernatant was extracted with equal volumes of phenol:chloroform:isoamyl alcohol (25:24:1, vol:vol:vol; Sigma Aldrich, United States), followed by extraction with equal volumes of chloroform: isoamyl alcohol (24:1, vol:vol; Sigma Aldrich, United States). DNA was precipitated with 1 ml polyethylene glycol 8,000 (Sigma Aldrich, United States) at 4°C overnight, centrifuged at 19,000 × *g* for 30 min. The precipitates were washed with ice cold 70 vol% ethanol solution, dried up in the air and then dissolved in 400 μl TE buffer (10 mM Tris, 1 mM EDTA, pH 8.0) and stored at -20°C before use.

Abundances of archaeal 16S rRNA genes were determined on LightCycler 480 (Roche, US) using the primer sets Arch 806F (*ATT AGA TAC CCS BGT AGT CC*) (Takai and Horikoshi, 2000) and Arch 958R (*YCC GGC GTT GAM TCC AAT*) (DeLong, 1992). A fragment covering the hypervariable regions (V3–V6) of the Archaeal 16S rRNA gene were PCR amplified using the primers Arch344Fmod (5′-GGGYGCAGCAGKCGMGAA-3′) and Arch915R (5′-GTGCTCCCCCGCCAATTCCT-3′) (Goodfellow and Stackebrandt, 1991). The forward primer was modified from the initial primer Arch349F (5′-GYGCASCAGKCGMGAAW-3′; Takai and Horikoshi, 2000) by extending the 5′-end and reducing degeneracy without losing coverage as determined by *in silico* tests using TestPrime 1.0 (Klindworth et al., 2013). PCR was carried out in 25 μl reaction volume with GeneAmp 9700 Thermal Cycler (Applied Biosystems, United States) with the following cycling parameters, initial pre-denaturation at 98°C for 2 min, followed by 25 cycles of denaturation at 95°C for 30 s, annealing at 60°C for 45 s, elongation at 72°C for 1 min, plus a final extension at 72°C for 10 min. The PCR mixture contained 0.125 μl TaKaRa ExTaq Hot Start polymerase (TaKaRa, Japan), 2.5 μl of 10× Ex Taq buffer, 2 μl dNTP solution (2.5 mM), 1 μl of each primer (10 μM), 2 μl BSA (10 μg μl^-1^), 1 μl of template DNA, and 15.375 μl de-ionized ultrapure water. In a second PCR, all PCR products were supplied with the forward and reverse Illumina^®^ adapter overhang sequences, which are provided in Illumina^®^ published “16S metagenomic sequencing Library preparation protocol”^1^. The resultant PCR products were purified and supplied with indices using the Illumina^®^ Nextera XT Index Kit. Negative controls without template were included to test for reagent contamination in each set of PCR reactions. PCR products were evaluated on 2% agarose gels, and purified using the Agencourt AMPure XP Kit (Beckman Coulter, Inc., United States) before used as template for the next PCR. The final purified PCR products were quantified by a Qubit 2.0 fluorometer with the dsDNA HS Assay Kit (Life Technologies, United States), and then pooled in an equimolar ratio. The pooled library was sequenced on an Illumina^®^ MiSeq system using a 600 cycle MiSeq v3 Reagent Kit (Illumina, United States), which produces two 300-bp long paired-end reads. An average of 39,026 (ranging from 16,477 to 66,068) reads were generated for all samples, and more than 99.7% of them classified as archaea, thus indicating good specificity of the primers.

### Quality-Filtering and Sequence Analysis

The paired-end MiSeq reads were processed using MOTHUR 1.36.1 (Schloss et al., 2009) by following the MiSeq SOP pipeline (Kozich et al., 2013). Merged reads with length shorter than 540 bp or longer than 599 bp, or homopolymeric stretches longer than 7 bp were all removed from the dataset in the initial quality filtering. Reads were further denoised by pre-clustering and the chimeric sequences were checked and filtered out by UCHIME (Edgar et al., 2011). The filtered sequences were aligned and classified based on the SILVA SSU REF NR v.123 database (Quast et al., 2013; Yilmaz et al., 2014). Sequences not aligning within the expected region flanked by the PCR primers were removed from further analysis. Methanogen taxonomic lineages were classified into different physiological categories depending on their presumed substrate preference (Canfield et al., 2005; Jabłoński et al., 2015): hydrogenotrophic (H_2_) using H_2_/CO_2_, acetoclastic (Acetate) using acetate, methylotrophic (Methyl) using non-competitive methylated substrates (MAs, methanol, etc.), mixotrophic (Mix) using more than one type of substrates, and unclassified (N). The sequences generated in this study were deposited in the NCBI Sequence Read Archive (SRA) under project no. PRJNA359637.

## Results

### Geochemical Profiles

**FIGURE 1:**
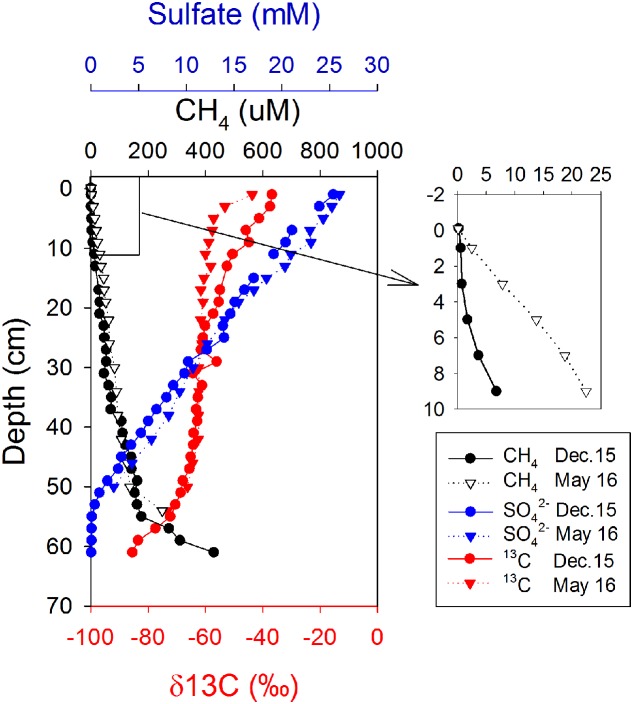
Depth distributions of sulfate, methane, and δ^13^C of methane on two sampling occasions.

Profiles of geochemical parameters showed similar pattern in cores from the two cruises in December 2015 and May 2016 (**Figure 1**). Methane pore-water concentrations increased with depth, from 0.6 to 51 μM in the top 20 cm, where the sulfate concentration was >15 mM. There was a slight gradient of CH_4_ from the sediment to the overlying seawater (**Figure 1**, enlarged section). The gradient was used to calculate diffusive methane fluxes across the sediment-water interface using Fick’s first law. The estimated methane flux was 0.22 nmol cm^-2^ d^-1^ during December and 2.13 nmol cm^-2^ d^-1^ during May (**Table 1**). This is a minimal estimate, as the effect of bioturbation was not taken into account. The sulfate–methane transition zone was shallow, at around 50 cm, but there was a long tailing of methane up through the sulfate zone. The δ^13^C of methane decreased with depth from -30‰ to -80‰ in both profiles, with a small shift to more negative values in the top 20 cm for the May samples.

**Table 1:** Depth-integrated (0–16 cm) rates of different processes in surface sediments of this study.

Processes (rates)	December 2015	May 2016
CH_4_ flux (nmol cm^-2^ d^-1^)	0.22	2.13
CH_4_ production (nmol cm^-2^ d^-1^)	1.00	0.83
CH_4_ oxidation (nmol cm^-2^ d^-1^)	1.11	0.93
Sulfate reduction (nmol cm^-2^ d^-1^)	553.53	623.34
DIC production (nmol cm^-2^ d^-1^)	–	1080.41


### Dynamics of Methane and Isotopes during Incubation

**FIGURE 2:**
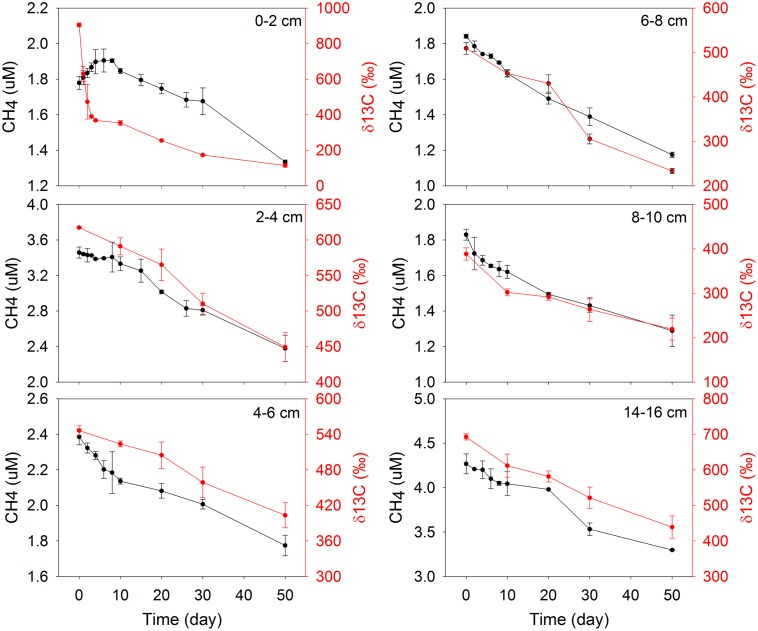
Concentration and ^13^C isotopic composition of CH_4_ in the bags during incubation of December samples.

The initial concentration of CH_4_ in each bag did not increase with depth as it did in CH_4_ profiles, which was due to loss of CH_4_ during slicing and sieving. During incubations, the concentrations of CH_4_ decreased with time for all depths except for the 0–2 cm (**Figure 2**). In this top section, CH_4_ increased from 1.7 to 1.9 μM over the first 4 days, and then decreased with a similar trend as at other depths. This is consistent with the idea that that some active substrates were available in the top 0–2 cm for methanogenesis, which became depleted rapidly in a few days. Therefore, at 0–2 cm the modeling and calculation of methane turnover rates was separated in two phases, 0–4 days and 6–50 days. The δ^13^C values decreased with time in all incubations (**Figure 2**), as evidence of methane production from a non-spiked carbon source (Seifert et al., 2006). Similar patterns of CH_4_ concentrations and δ^13^C values were displayed during bag incubations in May (Supplementary Figure S3).

### Rate Calculations Based on Isotope Dilution Model

**FIGURE 3:**
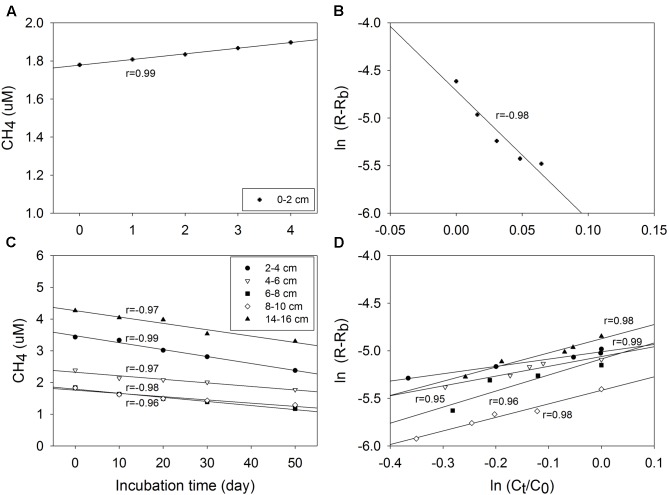
Plot and linear fitting of total CH_4_ concentrations (C_t_) and ^13^C-CH_4_ relative abundance (R) during incubation of sediment samples from December 2015: total CH_4_ (C_t_) concentration against incubation time **(A,C)**, and ln(R – R_b_) against ln(C_t_/C_0_) **(B,D)**, R_b_ is the mole fraction of ^13^CH_4_ in CH_4_ being produced from indigenous sources in the sediment.

**FIGURE 4:**
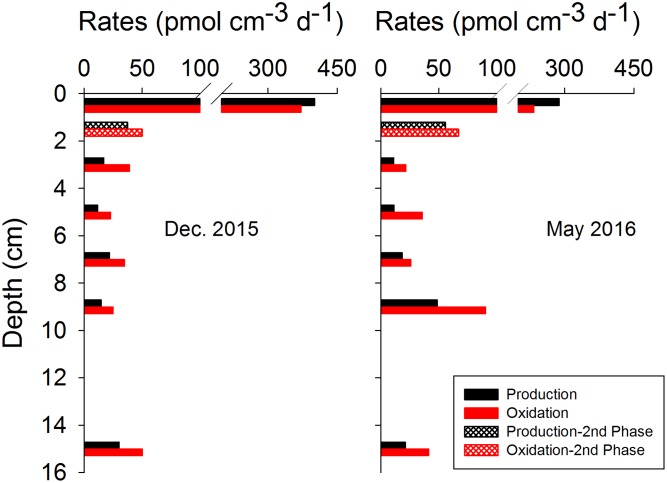
Depth distributions of CH_4_ production and oxidation rates in December 2015 (left panel) and May 2016 (right panel), as calculated from bag incubation data of total CH_4_ concentrations and ^13^C-CH_4_ relative abundance.

As assumed by the isotope dilution model, the correlation coefficients were all above 0.96 by linearly fitting CH_4_ concentrations with time (**Figure 3**). The validity of the model is more significantly corroborated by the linearity demonstrated by the plots of ln(R - R_b_) against ln(C_t_/C_0_), and the correlation coefficients were all above 0.95. Similar trends and good fitting were also observed in experiments repeated in May 2016 (Supplementary Figure S4), and in the second phase of 0–2 cm (Supplementary Figure S5). Based on the modeling data, production and oxidation rates of CH_4_ were calculated (**Figure 4**). Both production and oxidation rates peaked in the top layer with initial rates of over 200 pmol cm^-3^ d^-1^, and then decreased steeply to below 100 pmol cm^-3^ d^-1^ in all other layers, with no clear depth trend. Also during the second phase in 0–2 cm, rates were <100 pmol cm^-3^ d^-1^. The depth-integrated rates of CH_4_ production and oxidation were slightly higher in December 2015 compared to May 2016 (**Table 1**), which were all around 1 nmol cm^-2^ d^-1^ in 0–16 cm.

### Mineralization Rates

**FIGURE 5:**
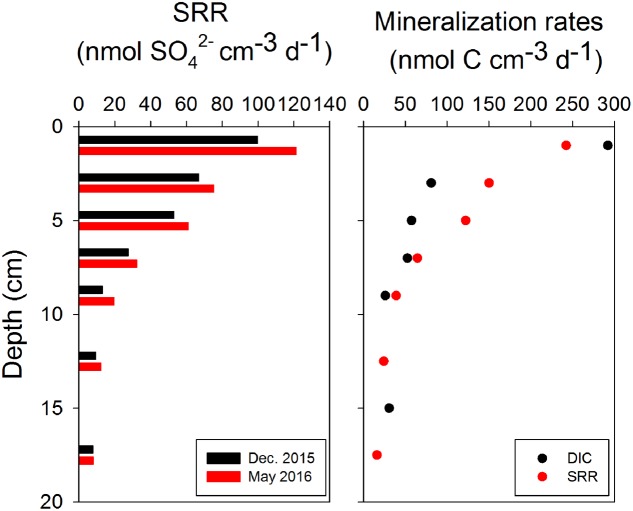
Sulfate reduction rates (SRR) in surface sediments from December and May cruises (left panel), and carbon mineralization rates calculated from production of DIC in bags and SRR in the core (1:2 stoichiometry between SRR and organic C oxidation rates) in May 2016 (right panel).

The highest SRRs of up to 100 nmol cm^-3^ d^-1^ were detected at 0–2 cm in December 2015, and then decreased gradually to below 20 nmol cm^-3^ d^-1^ at 17.5 cm (**Figure 5**, left panel). In May 2016, a similar distribution of SRR with depth was observed, while the SRR were slightly higher at each depth compared to December 2015 (**Figure 5**, left panel). The rates of organic carbon mineralization rates calculated from SRR gave values close to production rates of DIC (**Figure 5**, right panel, and Supplementary Figure S6), except at 0–2 cm, where the DIC production rate (290 nmol cm^-3^ d^-1^) was higher than the mineralization rate calculated only from SRR. The depth-integrated rates of SRR and carbon mineralization were two to three orders of magnitude higher than both flux and turnover rates of CH_4_ in 0–16 cm (**Table 1**).

### Methanogens in the Surface Sediment

**FIGURE 6:**
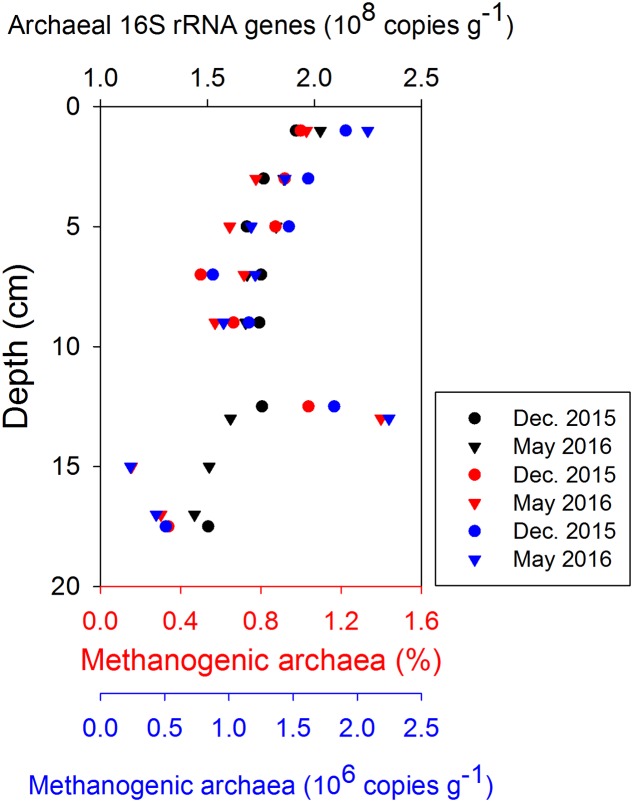
Depth distributions of archaea abundance (qPCR of 16S rRNA, in black), the relative abundance of methanogens (i.e., reads classified as methanogens/all archaeal reads, in red), and the absolute abundance of methanogens in the top 0–20 cm (estimated by multiplying their relative abundances with total archaeal 16S rRNA gene copies, in blue).

**FIGURE 7:**
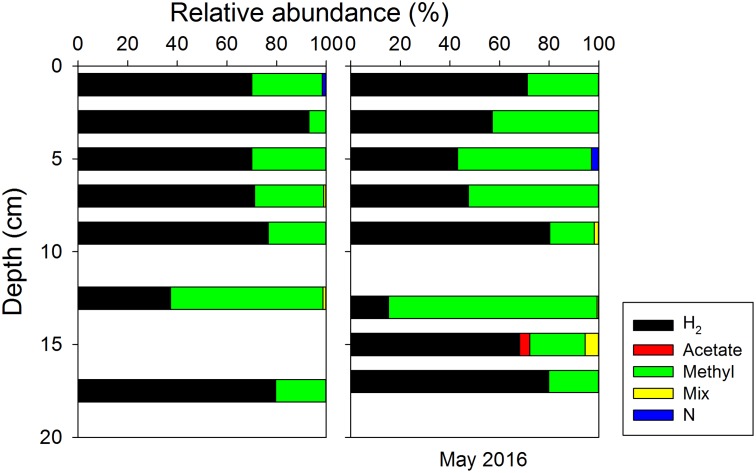
Depth distributions of different groups of methanogens classified according to presumed substrate usage: hydrogenotrophic (H_2_) using H_2_/CO_2_, acetoclastic (Acetate) using acetate, methylotrophic (Methyl) using non-competitive methylated substrates, mixotrophic (Mix) that may use more than one type of substrates, and unclassified (N).

The *in situ* archaeal communities and their depth distribution were similar at the phylum level during the two sampling occasions (Supplementary Figure S7), and dominated by Woesearchaeota, Euryarchaeota, Thaumarchaeota, and Bathyarchaeota (formerly known as the Miscellaneous Crenarchaeotal Group) (Meng et al., 2014). The relative abundances of Bathyarchaeota were constant with depth at around 10% of all Archaea, but no methanogens were classified into this phylum due to lack of taxonomy information at lower levels. Methanogenic archaea belonging to Euryarchaeota were present throughout the surface sediment, where their relative abundances (reads classified as methanogens/all reads) were generally less than 1.4% of all Archaea (**Figure 6**). Thus, the absolute abundances of methanogens, estimated by multiplying their relative abundances with total archaeal 16S rRNA gene copies, were two orders of magnitude lower than the total archaeal communities. Both the relative abundances and absolute abundances of methanogens decreased with depth, except at around 13 cm (**Figure 6**). Most methanogen-related sequences in the surface sediments belonged to unclassified Methanomicrobiales, *Methanococcoides*, and *Methermicoccus* (Supplementary Figure S8). The distribution of methanogens classified according to their inferred metabolic type is summarized in **Figure 7**, and hydrogenotrophic and methylotrophic methanogens dominated in all samples, while putative acetoclastic methanogens appeared only in a few samples with low abundances. A minor proportion of methanogens could not be assigned to a specific metabolic type.

## Discussion

Bag incubation of sediment combines the merits of slurry incubations with jars and whole-core incubations, and provides low heterogeneity close to natural conditions during anoxic incubation of sediment (Hansen et al., 2000). This bag incubation method has proven to be a useful tool in the study of anaerobic biogeochemical processes, such as degradation of organic matter, iron reduction, manganese reduction, and sulfate reduction (Canfield, 1989; Canfield et al., 1993a,b; Thamdrup and Canfield, 1996; Hansen et al., 1998, 2000). Here we extend this method to quantify CH_4_ turnover by combing it with an isotope dilution technique. For a gas like CH_4_, it is critical to avoid air pockets in the bag, as even a small headspace might cause a significant loss of CH_4_ from pore-water into the gas phase (Yamamoto et al., 1976). Our method calculates methane turnover rates during an incubation period, and thus averages out some finer temporal variations that may occur. Therefore, information about non-constant rates is lost, but few artifacts are created. The sieving of sediment before incubation and kneading of bag before each sampling aims to make sediment homogeneous in the bag, which allows repeated sampling during a time-course experiment (Hansen et al., 2000). However, these operations inevitably alter the structure of the original sediment, and may influence the rates of CH_4_ turnover and sulfate reduction, for example, influx of oxidants through bioturbation and bio-irrigation is blocked in bag incubations, which could stimulate sulfate reduction. Hansen et al. (2000) found that in the deeper part of sediment or those sediments with low oxygen penetration depth, whole-core and bag based SRR were quite close, while bag-rates exceeded whole-core rates by 1.4- to 3.2-fold in the more oxidized sediment. Thamdrup and Canfield (1996) found that carbon mineralization rates in bag incubations were close to rates from flux measurements in sediment core in continental margin sediments off central Chile, and Hansen et al. (1998) reported that anaerobic methane oxidation rates based on ^14^CH_4_ labeling in bag incubations were similar to rates in intact sediment core in Norsminde Fjord, Denmark. In our study, the sediment has a low oxygen penetration depth (Rasmussen and Jørgensen, 1992) and the majority of macro fauna were removed during sieving, which minimized the effect of degradation of entombed fauna (Canfield et al., 1993a), and hence we take the rates from bag incubation as estimates for *in situ* sediment metabolism. Our latest measurements in new samples from the same site confirmed that SRR and methanogenesis rates (based on ^14^CO_2_ labeling) determined in incubation bags were quite close to rates determined in intact sediment cores (Xiao et al., in preparation).

In spite of potential for methanogenesis in surface sediment which has been demonstrated by several earlier studies (Oremland et al., 1982; King, 1984; Mitterer et al., 2001; Maltby et al., 2016; Zhuang et al., 2016), methane production is seldom reflected in the geochemical profiles of CH_4_ due to a characteristic concave-up shape most of the time, which is thought to be an indication of diffusion combined with net CH_4_ oxidation (Iversen and Blackburn, 1981; Alperin et al., 1988). The closely coupled CH_4_ production and oxidation in surface sediment in our study (**Figure 4**) may partially explain this. Methane production almost balanced methane oxidation so that a cryptic cycling of CH_4_ existed, leaving little imprint in the pore water chemistry. One of the general signatures of biogenic CH_4_ is that δ^13^C values are more negative than -50‰ (Reeburgh, 2007), yet the δ^13^C values were around -30‰ in the top 2 cm of sediment in the present study (**Figure 1**), elevated rates of CH_4_ oxidation obviously contributed to this enriched ^13^C. The substrate limitation effects on the δ^13^C of CH_4_ locally produced may play a role too, which was found in hypersaline microbial mats, and can be reversed by organic matter addition (Oremland et al., 1982; Kelley et al., 2014, 2015). The bag incubation method combined with the isotope dilution technique used here makes it possible to estimate CH_4_ production and oxidation rates simultaneously (Blackburn, 1979; Andersen et al., 1998; von Fischer and Hedin, 2002), which makes it possible to directly quantify CH_4_ turnover rates close to *in situ* conditions. The methanogenesis rates in surface sediment from Aarhus Bay are comparable to those quantified by ^14^C-labeled substrates in the shallow sediments of the Sonora Margin cold seeps, where rates peaked at 617 and 333 pmol cm^-3^ d^-1^, respectively in the top 7 cm of two sites, and then decreased with depth (Vigneron et al., 2015). In sediments in the Peruvian margin with low-oxygen conditions and frequent input of fresh organic matter, net methanogenesis rates of 0.03–0.1 mmol m^-2^ d^-1^ over 0–25 cm (Maltby et al., 2016) was found by measuring CH_4_ increase in headspace of incubation vials, corresponding to averages of 0.12–0.4 nmol cm^-3^ d^-1^.

Methane production rates peaked at 0-2 cm depth and dropped below as did SRR and organic matter mineralization rates (**Figures 4**, **5**), suggesting a pool of more degradable organic substrates near the sediment surface. However, thermodynamically, sulfate reducers are expected to outcompete methanogens in the sulfate-rich sediment for common substrates like acetate and hydrogen (Lovley and Goodwin, 1988; Hoehler et al., 1998). An enhanced CH_4_ production in the top layer may be unexpected because SRRs are also high in this layer (**Figure 5**). A possible explanation is that methylotrophic (non-competitive) methanogens could use non-competitive substrates like MA, DMS, and methanol as the dominant pathway (Oremland et al., 1982; King, 1984; Mitterer et al., 2001; Mitterer, 2010; Vigneron et al., 2015; Zhuang et al., 2016). These small organic compounds are ubiquitous in the marine environment, and originate from the decomposition of substances like choline, creatine and betaine, lignins and pectin, or from bacterial reduction of TMA oxide (Yancey and Somero, 1980; Oremland et al., 1982; Alcolombri et al., 2015). The downcore distribution of a large exchangeable pool of TMA (0.3–6.5 μmol kg^-1^) at the same site as this study implied an endogenous source of this substrate. The free pools of methylated substrates in the pore water were extremely low, however, with only a few nanomolar DMS (DMSP) and no detectable (<20 nM) free TMA in the pore-water (Zhuang, 2014). Yet, methanogenesis from these compounds with so low concentrations was still energetically favorable (Zhuang, 2014; Zhuang et al., 2016). The high affinity of some of these methylated compounds to solid phase (Wang and Lee, 1990, 1993) and the limitation of detection techniques make it difficult to quantitatively estimate their contribution to methanogenesis directly by experiments with ^14^C-labeled substrates due to poorly defined concentrations in the pore-water and potential exchange of radiotracer between free and adsorbed pools.

The correlation of microbial community profiles with geochemical data has been challenged by factors like limited datasets, low spatial resolution, and insufficient depth of the taxonomic profiling (Jørgensen et al., 2012). In our study, highest CH_4_ production rates co-occured with highest methanogen abundance at 0–2 cm depth. However, the high proportions of hydrogenotrophic methanogens, together with the methylotrophic methanogens in sulfate-rich sediments, deviated from our original hypothesis that methylotrophic methanogens should dominate there. We do not know whether these hydrogenotrophic methanogens were actually active or not. The potential methylotrophic methanogens (e.g., uncultivated members of the phylum Bathyarchaeota) also remain to be characterized and verified, though environmental genomics have indicated their potential for methanogenesis (Evans et al., 2015; Du Toit, 2016; Lever, 2016; Nobu et al., 2016; Vanwonterghem et al., 2016). On the other hand, the presence of hydrogenotrophic methanogens in the uppermost sediment might indicate that methanogens could still produce CH_4_ through this pathway even when the sulfate concentration is high, which has been demonstrated in moving bed biofilm reactors (Yang et al., 2015). These methanogens may have benefited from the heterogeneous and fluctuating redox conditions generated by bioturbation in the surface sediment, where sulfate reducers cannot effectively inhibit hydrogenotrophic methanogens through substrate competition (Kristensen et al., 2012; Chen, 2015). It is also possible that hydrogen leakage from methylotrophic methanogens enhanced hydrogenotrophic methanogenesis, a syntrophy which was previously found by co-culturing of a sulfate-reducer with hydrogenotrophic methanogens and methylotrophic methanogens (Finke et al., 2007; Ozuolmez et al., 2015).

The depth decrease of CH_4_ oxidation rate in the present study was similar to earlier research in the near-by Kysing Fjord in July (Iversen and Blackburn, 1981). Those authors directly quantified CH_4_ oxidation rates by determining the production of ^14^CO_2_ from injected ^14^CH_4_ during sediment incubations, and found the highest CH_4_ oxidation rate of ∼180 pmol cm^-3^ d^-1^ in 0–2 cm. In our study, the sediment was sectioned and sieved in an anaerobic glove box under a nitrogen gas atmosphere. Oxygen was therefore depleted in these sediments during the processing and stabilization of bag incubations, and anaerobic carbon mineralization should dominate in these bags (Thamdrup and Canfield, 1996). Sulfate was probably the most important electron acceptor due to its high concentration, and high activity of sulfate-reducing microbes in the sediment (**Figure 5**, left panel), however, the difference between DIC production rates and SRR-related mineralization rate in the top 2 cm (**Figure 5**, right panel) indicated that other electron acceptors like nitrate or ferric iron, which were not directly quantified in the present study, might also play a role in this layer. Nevertheless, we can still speculate that nitrate and iron-dependent anaerobic methane oxidation, which are thermodynamically favorable and for which microbial players have been found (Ettwig et al., 2010, 2016; Timmers et al., 2017), might have also been relevant during our bag incubations. The methane turnover rates over 0–16 cm were less than 0.2% of the total carbon mineralization rates, but the integrated rates over 0–16 cm were comparable to the CH_4_ flux rates calculated from CH_4_ profiles (**Table 1**), which was also shown in Maltby et al. (2016), implying that the methane turnover in surface sediment may play a more important role in CH_4_ exchange between sediment and above water than previously thought.

## Conclusion

Methanogenesis was found in marine surface sediment, which so far has eluded direct observation of methane profiles. This methane production was closely coupled to methane oxidation, leading to a cryptic methane cycling in marine surface sediment. Considering the common environmental conditions found in other coastal systems, we speculate that such a cryptic methane cycling can be ubiquitous but further work is needed to elucidate the role of different groups of methanogens and methanotrophs involved.

## Author Contributions

NR-P, BJ, and K-QX designed the study; K-QX and FB collected the sample, did the geochemical measurement, and analyzed the data; K-QX and KK did the molecular analysis; K-QX wrote the paper; all co-authors reviewed the paper.

## Conflict of Interest Statement

The authors declare that the research was conducted in the absence of any commercial or financial relationships that could be construed as a potential conflict of interest.

## Footnotes

1 http://support.illumina.com
