# Supplementary material for: Concurrent Methane Production and Oxidation in Surface Sediment from Aarhus Bay, Denmark
Source: Front Microbiol. 2017 Jun 30;8:1198. doi: 10.3389/fmicb.2017.01198 (PMC5492102; doi:10.3389/fmicb.2017.01198)
Supplement: Supplementary file 1 [file Data_Sheet_1.DOCX]

**Concurrent methane production and oxidation in surface sediment from Aarhus Bay, Denmark**

Ke-Qing Xiao*, Felix Beulig, Kasper Urup Kjeldsen, Bo Barker Jørgensen, Nils Risgaard-Petersen

Center for Geomicrobiology, Department of Bioscience, Aarhus University, Denmark

* Corresponding author

Address: Ny Munkegade 116, 8000 Aarhus C, Aarhus, Denmark.

Phone: +45-87156587

Email: .xiaokeqing12@bios.au.dk

**Materials and Methods**

**The Isotope dilution model**

Here we derive the equations used to calculate concurrent production and consumption of CH_4_ from isotope dilution according to Blackburn (1979).

The model assumes that both the rate of CH_4_ production, p, and the rate of consumption, r, are constant during the experiment and that both ^13^C-CH_4_  and ^12^C-CH_4_ are produced and consumed during the incubation. The change in methane concentration, C, with time can then be described as:

dC/dt = p-r (1)

C_t_ = C_0_+(p-r)t (2)

Here, C_0_ is the initial concentration. The change in ^13^C-CH_4_ and ^12^C-CH_4_ concentration with time can be described as:

d^13^C/dt = pR_b_-rR (3)

d^12^C/dt = p(1-R_b_)-r(1-R) (4)

Here R_b_ is the naturally occurring relative abundance (mol fraction) of ^13^C-CH_4_, and R is the relative abundance of ^13^C-CH_4_ in the bag, which can be measured during the incubation and is defined as:

R =^13^C /(^13^C +^12^C) (5)

Here ^13^C and ^12^C are the concentrations of ^13^CH_4_ and ^12^CH_4_ in the sediment at a given time.

With equation 3 and 5 we have:

dR/dt = (R_b_-R)p/C (6)

Combining equation 2 and 6, given that at time zero, C_t_= C_0_ and R= R_0_, we get:

ln(R_t_-R_b_) = ln(R_0_-R_b_)-(p/(p-r))ln((C_t_/C_0_) (7)

Eqution 7 assumes that p ≠ r, in the case of p = r, C_t_=C_0_, equation 6 can be simplified as:

dR/(R_b_-R) = p/C_0_ dt (8)

from which we can get,

ln (R_t_-R_b_) = ln (R_0_-R_b_) – p/C_0_t (9)

**References**

Blackburn TH (1979). Method for measuring rates of NH_4_^+^ turnover in anoxic marine sediments, using a ^15^N-NH_4_^+^ dilution technique. Appl Environ Microb 37: 760-765.
