# Supplementary figures and images for: Concurrent Methane Production and Oxidation in Surface Sediment from Aarhus Bay, Denmark
Source: Front Microbiol. 2017 Jun 30;8:1198. doi: 10.3389/fmicb.2017.01198 (PMC5492102; doi:10.3389/fmicb.2017.01198)

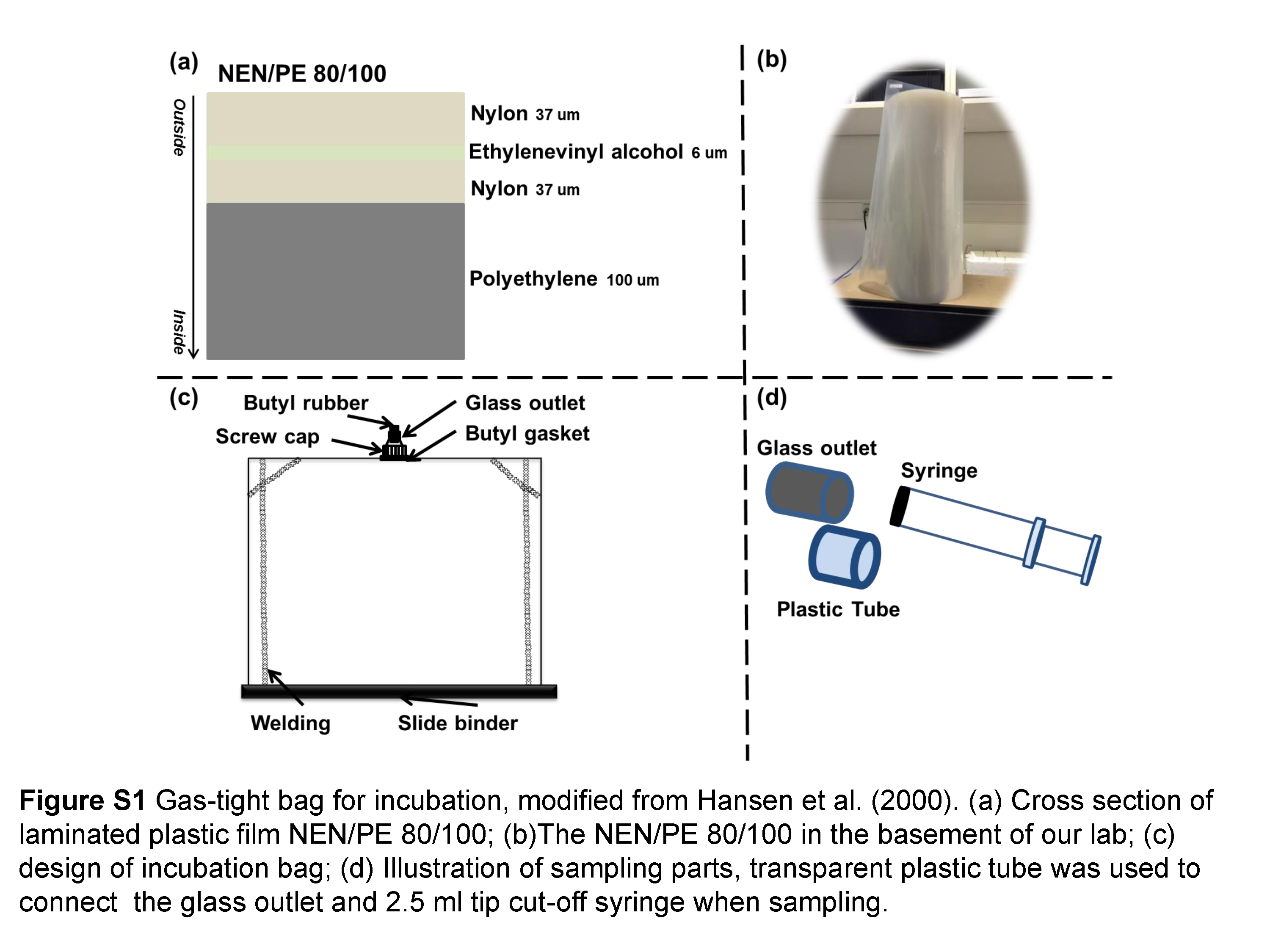

Supplement: Supplementary file 2 [file Image_1.TIFF]

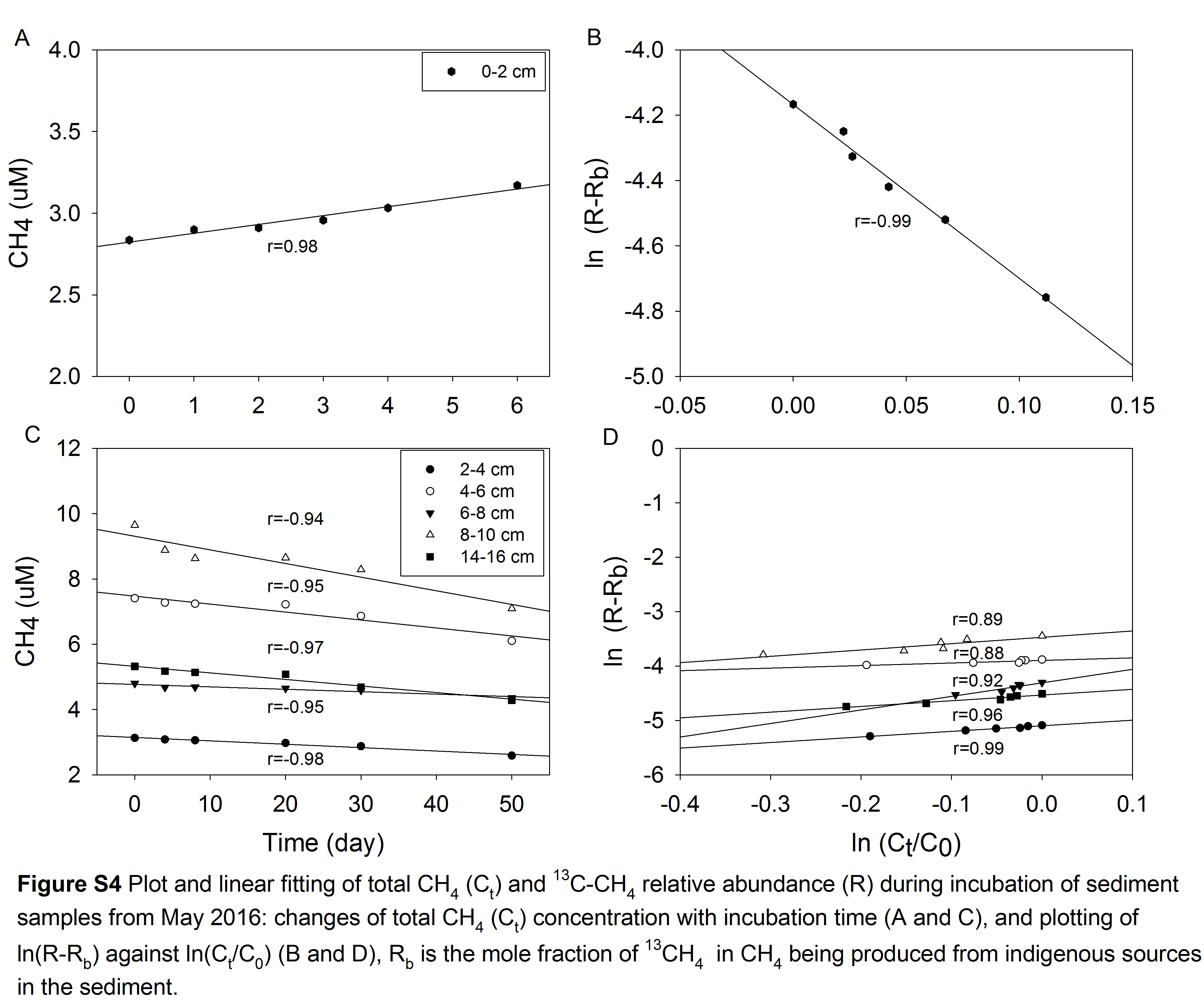

Supplement: Supplementary file 5 [file Image_4.JPG]
